# Latest Clinical Evidence and Operative Strategy for Small-Sized Lung Cancers

**DOI:** 10.14789/jmj.JMJ21-0030-OT

**Published:** 2022-01-21

**Authors:** ARITOSHI HATTORI, KENJI SUZUKI

**Affiliations:** 1 Department of General Thoracic Surgery, Juntendo University School of Medicine, Tokyo, Japan; 1 Department of General Thoracic Surgery, Juntendo University School of Medicine, Tokyo, Japan

**Keywords:** lung cancer, ground-glass opacity, surgery

## Abstract

Many thoracic surgeons revealed that consolidation tumor ratio or solid component size on thin-section computed tomography has been considered more prognostic than maximum tumor size in non-small cell lung cancer (NCSLC). According to the results, the 8^th^ TNM classification drastically changed the staging system, i.e., clinical T category was determined based on the invasive or solid component size excluding a ground-glass opacity (GGO). However, several debates are arising over the application of radiological solid size for the clinical T staging. Meanwhile, recent several institutional reports have noticed a significantly simple fact that the presence of a GGO denotes an influence on the favorable prognosis of NSCLC. More important, radiologic pure-solid lung cancers without a GGO exhibit more malignant behaviors with regard to both the clinical and pathological aspects, and show several histologic types that have a poorer prognosis than radiologic part-solid lung cancer. In contrast, favorable prognostic impact of the presence of a GGO component was demonstrated, which was irrespective of the solid component size in cases in which the tumor showed a GGO component. Recently, this concept has been gradually noticed on a nationwide level.

Obvious distinctions regarding the several baseline characteristics between the tumor with/without GGO component is a fundamental biological feature of early-stage lung cancer, which would result in a big difference in prognosis, modes of recurrence, overall behavior, and appropriate operative strategies. As a future perspective, the presence or absence of a GGO should be considered as an important parameter in the next clinical T classification.

## Introduction

Since the Japan Clinical Oncology Group (JCOG) study prospectively validated the radiological definition that enabled prediction of the pathological noninvasiveness of clinical stage IA lung cancer based on the findings of thin-section computed tomography (CT)^1)^, many thoracic surgeons have revealed that consolidation tumor ratio (CTR) and solid component size were more prognostic than maximum tumor size for resected non–small cell lung cancer (NCSLC)^2-6)^. This finding is extremely important in the history of general thoracic surgery. Subsequently, the 8^th^ edition of the TNM staging system drastically changed the staging system, with the clinical T category being determined according to solid component size and excluding ground-glass opacity (GGO)^7)^. In contrast, new issues are emerging from the proposed changes concerning T parameters. Much of the confusion is caused by the absence of a consensus on how to make uniform the measurements of solid component size in many part-solid tumors in which solid component size is difficult or impossible to measure^8, 9)^. In such circumstances, we have reported a new and simple fact that the presence of a GGO denotes a great influence on the favorable prognosis of NSCLC, and the radiological solid component size is irrelevant to the survival outcome of NSCLC if the tumors show a GGO component^10-17)^. On the other hand, radiologically determined pure-solid lung cancers without a GGO component exhibit more malignant behavior and show several histologic types that have a poorer prognosis than do radiologically part-solid lung cancers. Thus, the prognostic impact of the solid tumor size is considered to be meaningful only in the pure-solid NSCLC^10-15)^. This fact is extremely important when considering future revision of the clinical T staging and the proper operative strategies of lung cancer, provided that the clinicopathologic and oncologic outcomes are disparate between part-solid and pure-solid tumors on the basis of a GGO presence. In this report, we would like to demonstrate the latest clinical evidence regarding the small-sized lung cancer, and to discuss the appropriate operative modes based on these clinical evidences.

## Latest clinical evidence of small-sized lung cancer

**Figure 1:**
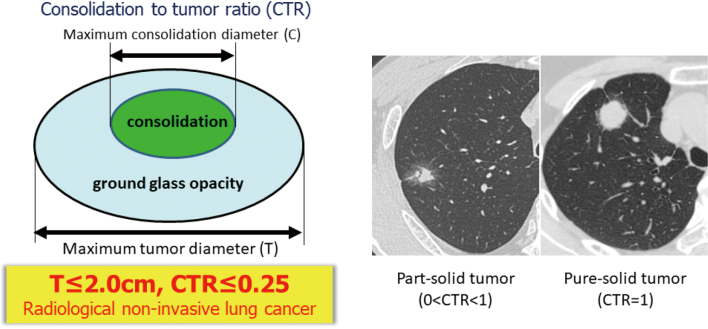
Definition of the consolidation to tumor ratio, and the typical findings of part-solid tumor and pure-solid tumor based on thin-section computed tomography^1)^.

To date, there are numerous studies to evaluate the radiological and pathological correlation of early-stage NSCLC in Japan^18-20)^. Based on the findings of thin-section CT scan, small-sized lung cancer is radiologically comprised of 2 parts, which is consolidation part and ground-glass opacity component^1)^. Ground-glass opacity, or GGO is defined as an area of a slight, homogenous increase in density that do not obscure the underlying vascular marking (Figure 1). When the tumor is surrounded by a GGO component, it is called as part-solid tumor with a GGO. By contrast, pure-solid tumor is recognized as a tumor without any GGO component^12, 16, 21)^ (Figure 1). And it is well known that the ratio of consolidation part to the maximum tumor size well reflects the tumor aggressiveness in early-stage lung cancer^1)^. When we defied a consolidation to tumor ratio (CTR), which indicates the ratio of maximum consolidation diameter to the maximum tumor diameter, tumor size less than 2cm and CTR less than 0.25 was defined as a radiologically non-invasive lung cancer to predict pathological non-invasiveness based on the result of JCOG0201 trial^1)^. Furthermore, the 5-year survival outcome was significantly different when the cutoff point of CTR was selected as 0.5 (CTR≤0.5, radiological non-invasive; 96.7%, CTR>0.5, radiological invasive; 88.9%, p<0.001)^1, 22)^. However, even in the radiological invasive lung cancer with a CTR more than 0.5, recent study shows that the presence of a GGO component has a strong impact on the favorable prognosis of lung cancer^10, 13, 17)^.

**Figure 2:**
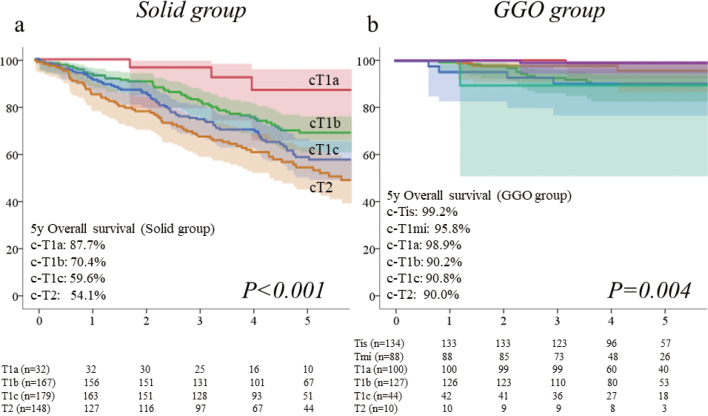
Clinical T category was compared in the GGO and Solid groups, respectively. The 5y-OS was excellent being 90% or more despite the revised T categories, provided the tumor had a GGO appearance. In contrast, maximum tumor size significantly separated the OS in the Solid arm (p<0.001)^12)^.

Until now, we focused on the clinicopathological and prognostic importance of a GGO component from several aspects. In general, we have reported that the lung cancer with a GGO component showed less invasive feature compared to the radiological pure-solid tumor. For instance, among patients with clinical-stage IA disease with a radiological invasive appearance (i.e., CTR>0.5), the frequency of pathological nodal metastasis is quite distinct based on the presence of GGO component, which is approximately estimated that the pathological nodal metastasis is found in 3-5% of part-solid lung cancer (0.5<CTR<1.0), but 15-20% of pure-solid lung cancer (CTR=1.0)^14)^. Furthermore, among the c-stage IA radiological invasive lung cancer (CTR>0.5), the prognosis is significantly different between the part-solid tumor with GGO and the pure-solid tumor without GGO, and the survival differences were never shown in radiological invasive NSCLC with GGO component (0.5< CTR<1.0), which was regardless of solid component size or CTR^10, 13, 17)^. All the more, the prognosis is quite excellent showing more than 90% in 5y overall survival (OS), if tumor has a GGO component^10-13, 15, 23, 24)^. In contrast, radiologically determined pure-solid lung cancers without a GGO component exhibit more malignant behavior and show several histologic types that have a poorer prognosis than do radiologically part-solid lung cancers. Furthermore, as shown in our previous study (Figure 2), the prognostic impact of the tumor size is considered to be meaningful only in the pure-solid NSCLC^10-13, 15, 23, 24)^. That is, the survival curves split almost fairly among the different solid component sizes only in radiological pure-solid lung cancer. This fact is extremely important when considering future revision of the clinical T staging of lung cancer, provided that the clinicopathologic and oncologic outcomes are disparate between part-solid and pure-solid tumors on the basis of a GGO presence.

## Proposal for novel clinical T staging

**Figure 3:**
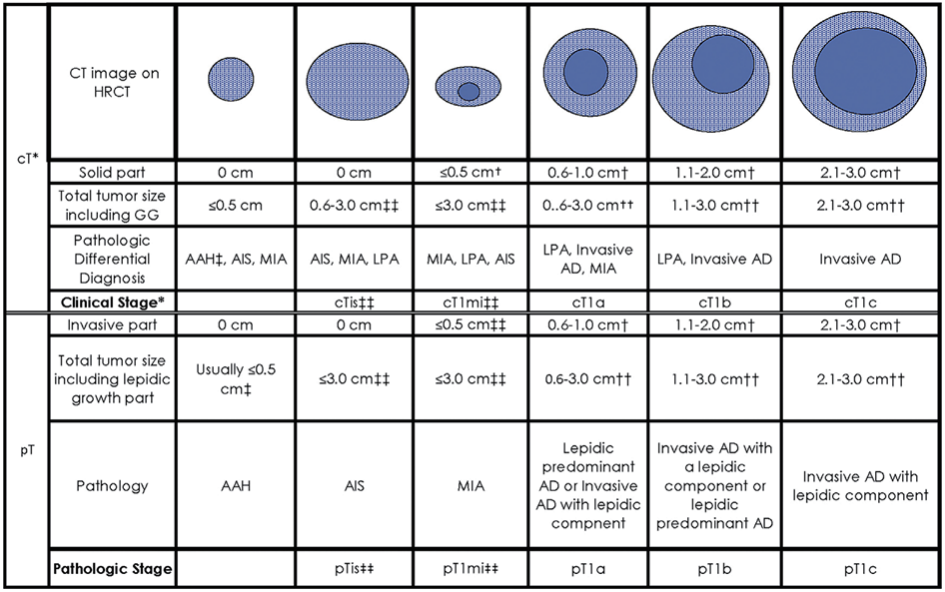
Proposed 8th edition of the cT and pT descriptor classifications of small lung adenocarcinomas with a GGO and lepidic component by computed tomography and pathologic diagnosis^7)^.

**Figure 4:**
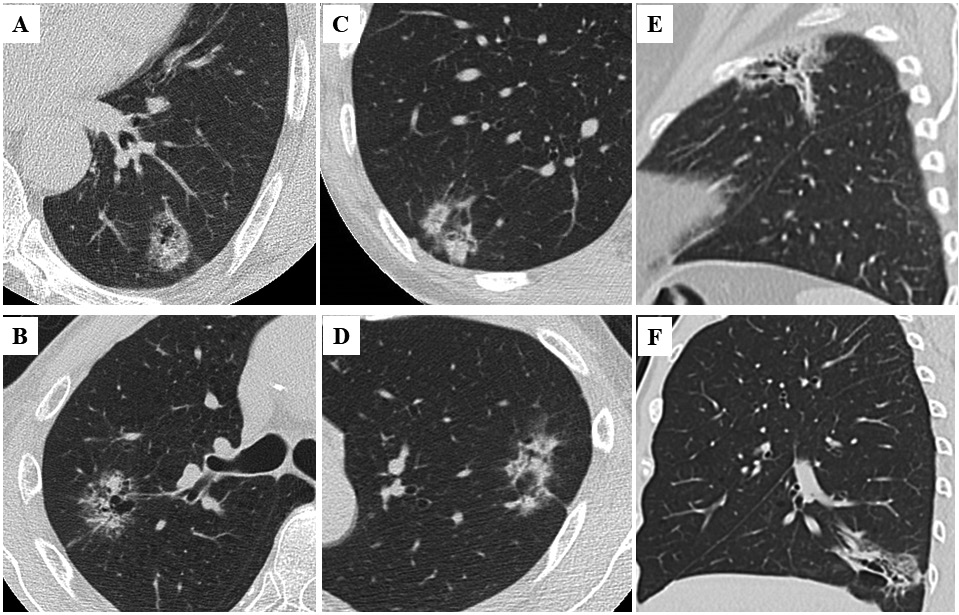
Radiological findings of part-solid lung adenocarcinomas with a solid component size that is difficult to measure: GGO with scattered consolidations (A, B), GGO with island shaped consolidations (C, D), or GGO mimicking organizing pneumonia (E, F).

Based on the latest clinical evidences, we are rigorously proposing a proper lung cancer staging for the next clinical T classification. Currently, solid component size is used as a clinical T factor based on the 8^th^ edition of TNM staging system^7)^ (Figure 3). However, several issues are arising regarding the application of the solid component size as a clinical T staging. At first, inconsistency exists between radiological solid component size and pathological invasive size in part-solid lung adenocarcinomas, because the solid area often represents a benign scar or a fibrous scar harboring a stromal invasive component in part-solid tumors^25, 26)^. Furthermore, there are several findings of part-solid tumors in which the solid component size is quite difficult or impossible to measure due to the presence of multiple, complicated or scattered solid areas rather than a single focus^8, 9, 27)^, which has not been absolutely determined in the new proposal. In the 8th edition of the T classification, there is no consensus on how to make uniform the measurements of solid component size in many part-solid tumors^7)^. These include not only typical GGO-dominant or solid- dominant part-solid tumors including multifocal expression, but atypical part-solid lesions such as GGO with scattered consolidations^9)^ (Figure 4A and 4B), GGO with island shaped consolidations^28, 29)^ (Figure 4C and 4D), or GGO mimicking organizing pneumonia^27)^ (Figure 4E and 4F).

All the more, there exists more critical and fundamental issue in the classification of lung cancer staging. Based on the previous background, we always deem whether it is necessary to classify the prognosis of part-solid lung cancer with a GGO based on the solid component size. Again, clinicopathological and oncological features are significantly different between part-solid tumor with GGO and solid tumor without GGO. Therefore, we proposed to classify the current T staging to the subgroup based on the presence or absence of GGO (i.e., GGO group and Solid group). As a result, the 5y-OS was distinct in pure-solid tumor without GGO, which was worse based on the tumor size. However, the prognosis of part-solid tumor with a GGO component was not significantly different regardless of the solid component size, and their prognosis was excellent, which was irrespective of the current T staging^12)^ (Figure 2). This fact is extremely important when considering future revision of the clinical T staging of lung cancer, provided that the clinicopathologic and oncologic outcomes are disparate between part-solid and pure-solid tumors on the basis of a GGO presence.

Recently, this concept has been gradually noticed not only in Japan but several other countries^30-35)^. Despite single institution advocacy for the prognostic importance of the presence of a GGO component as a significant clinical T parameter, however, this notion has not been fully confirmed across institutions or at a nationwide level. To validate this fundamental and simple prognostic feature of lung cancer, we aimed to demonstrate the prognostic impact of the presence of a GGO component in clinical stage IA NSCLC based on the long-term follow-up data of JCOG0201^15)^. As a result, significant center validation also suggested the favorable prognostic impact of the presence of a GGO component in the prospective JCOG0201 dataset.

**Figure 5:**
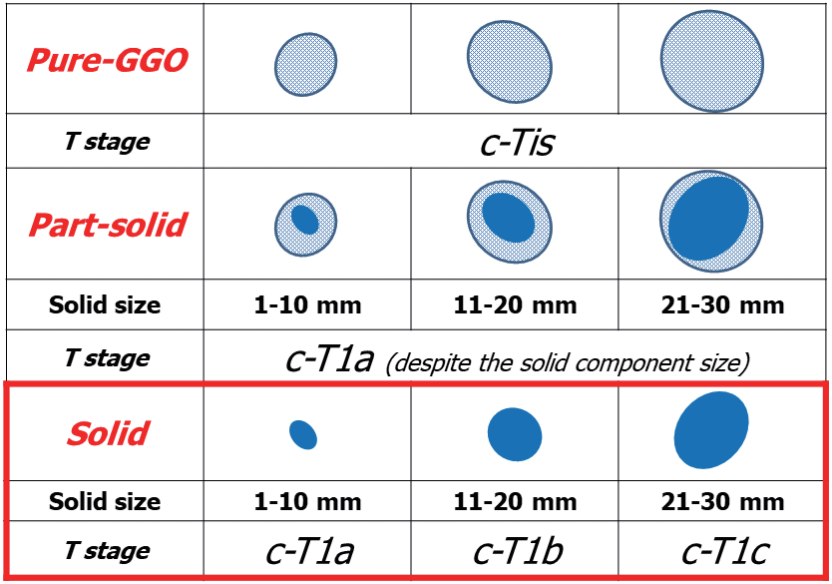
Proposed clinical T category based on the presence of a GGO component^11, 36)^.

The principle behind the TNM staging system is the classification of cancers into groups according to the anatomic extent. This contributes to evaluate treatment strategies and to give some indication of prognosis for survival. Hence, precise measurement of tumor size is crucial to improve stratification of lung cancer in the future. Based on these clinical backgrounds, we propose a new clinical T staging based on a GGO component in many reports, because we believe that the T staging should be simple, useful and reproducible to reflect the prognosis of lung cancer. Based on our clinical research, 5y-OS of part-solid lung cancer with GGO was more than 90% regardless of whole tumor size or solid component size. Therefore, we believe that part-solid lung cancer could be demonstrated as c-T1a despite their tumor size. In contrast, Tumor size has a great impact on the prognosis only in radiological pure-solid lung cancer. Therefore, tumor size effect should be exclusively applied to the radiological solid lung cancer without GGO component (Figure 5)^12, 36)^.

## Appropriate operative strategy for small-sized lung cancers

Today, standard operative mode for resectable lung cancer is recognized as lobectomy, this is based on the evidence from USA in 1995^37)^. This randomized trial evaluated the OS of lobectomy and limited resection for clinical T1 non-small cell lung cancer. As shown in the report by Lung Cancer Study Group, lobectomy conferred limited or sublobar resection in both overall survival and recurrence-free survival (RFS). And the rate of locoregional recurrence of limited resection was 3-times higher than lobectomy. Hence, it is considered that the great caution is needed to indicate the limited pulmonary resection for lung cancer. However, due to the advancement of thin-section CT scan, more and more small-sized lung cancer was detected. Furthermore, based on the radiological and pathological correlations, we can predict pathologically less invasive tumor based on the radiological features. Currently, one of the most important prognostic factors would be a presence or absence of a GGO component, as presented in this lecture. Hence, it is possible to change the paradigm of standard operative strategy in a future^16)^.

Here, I would like to introduce 2 important trials conducted in Japan. These studies have been performed by JCOG lung cancer study group. The first trial presents a phase II study to evaluate the feasibility of wide wedge resection for GGO-dominant lung cancer, JCOG0804 trial^38)^. The second trial present a phase III study to evaluate the survival outcomes between segmentectomy and lobectomy for radiologically invasive lung cancer^39)^. At first, with regard to the feasibility study to evaluate the wedge resection for GGO-dominant or less invasive lung cancer, the 5y-RFS was 99.7%, which was quite excellent result^38)^. Hence, it is considered that the sublobar resection, preferably wedge resection, is enough for GGO-dominant lung cancer. Furthermore, the result of survival outcome for radiologically invasive lung cancer will be disclosed near future. We should carefully await this randomized trial of segmentectomy compared to lobectomy in radiologically invasive small-sized lung cancer to consider the appropriate operative modes of small-sized peripherally located NSCLC. However, at the standpoint of tumor invasiveness, there exist several controversies regarding the appropriate operative strategy for radiological invasive lung cancer. In particular, as introduced in many times in this lecture, radiological pure-solid lung cancer without GGO component shows aggressive invasive nature, and we have reported the higher frequencies of locoregional recurrence after segmentectomy for clinical-stage IA radiological pure-solid lung cancer^40, 41)^. With regard to the proper operative modes for peripherally located small-sized lung cancer, the result of randomized trial of segmentectomy compared to lobectomy is awaited, however the indication of segmentectomy or limited surgical resection for radiological pure-solid tumor should be in great caution from the point of cancer control.

## Conclusions

In this lecture, we demonstrated the latest clinical evidence and the operative strategies for small-sized lung cancers. In early-stage lung cancer, it has been clarified that the presence of a GGO is strongly contributed to the oncological aggressiveness and prognosis. This indicates that not a solid component, but a presence of GGO in itself is a matter of concern regarding the prognosis of small-sized lung cancers. Based on the clinical evidences, lung adenocarcinoma with a GGO component is deemed as a favorable clinicopathologic subgroup different from the pure-solid tumor. Clinical T staging should be classified based on the presence of a GGO, and tumor size be applied only to the radiological solid tumor. With regard to the operative strategies for small-sized lung cancer, it is awaited the result of randomized trial of segmentectomy compared to lobectomy in radiologically invasive small-sized lung cancer, however, it should be fully deliberate regarding the indication of limited surgical resection for radiological pure-solid tumor.

## Funding

This work was supported in part by a Grant-in-Aid for Cancer Research from the Ministry of Health, Labour and Welfare, Japan, the Smoking Research Foundation, and the National Cancer Center Research and Development Fund (26-A-4).

## Author contributions

AH performed the manuscript conceptualization, data curation, formal analysis, investigation, and writing of the original draft. KS contributed to the manuscript conceptualization, supervision and review & editing of the original graft.

## Conflicts of interest statement

We have no conflict of interest to disclose.
